# Dr. Fell the American Cancer Doctor

**Published:** 1857-10

**Authors:** 


*Dr. Fell, the American Cancer doctor.*—The name of Dr. Fell has been of late bruited in our newspapers in connection with the case of the distinguished American sculptor, Crawford, who has been under his treatment, in

London, for a malignant disease situated behind the orbit. This Dr. Fell, it appears, is a graduate in medicine of the New York University, who settled in London and professed to be able to cure carcinoma by means of a local application used by the North American Indians on the shores of Lake Superior! The managers of Middlesex Hospital, with more liberality than wisdom, placed at his disposal a ward for cancerous patients, he promising to make known the remedy used at the end of six months. This period having expired, the cancer doctor has reluctantly, as it seems, been compelled to publish a recipe of the application. It turns out to be chloride of zinc in combination with the root of the sanguinaria canadensis. Both have been for a long time, as our readers need not be informed, used as escharotics.

We have in this instance another illustration of the folly of encouraging a trial of remedies which are to be kept secret till their efficacy is established. The cancer doctor succeeded in obtaining the sanction of a well known hospital, and this was all he desired. In the mean time, he has no doubt filled his pockets with fees from credulous paying patients. The managers of Middlesex Hospital are probably now ready to echo the sentiment contained in the well known lines, "I do not like thee Dr. Fell," etc. *

